# Paraplegia Caused by IgG4-Related Hypertrophic Pachymeningitis With an Elevated Cerebrospinal Fluid Immunoglobulin G4 Concentration

**DOI:** 10.7759/cureus.114251

**Published:** 2026-08-10

**Authors:** Hisaki Doyama, Yasuko Matsumoto, Akiho Kurosaka, Motoya Kobayashi, Kazuyoshi Yamaguchi

**Affiliations:** 1 Department of Neurology, Ishikawa Prefectural Central Hospital, Kanazawa, JPN; 2 Department of Orthopaedic Surgery, Ishikawa Prefectural Central Hospital, Kanazawa, JPN

**Keywords:** cerebrospinal fluid biomarkers, hypertrophic pachymeningitis, igg4-related disease (igg4-rd), igg4-related spinal pachymeningitis, spinal paraplegia

## Abstract

Immunoglobulin G4-related hypertrophic pachymeningitis (IgG4-RP) is a rare manifestation of immunoglobulin G4 (IgG4)-related disease and an uncommon cause of hypertrophic pachymeningitis (HP). Because serum IgG4 concentration is not necessarily elevated, the diagnosis of IgG4-RP is often challenging. We report a rare case of IgG4-RP in a 78-year-old woman presenting with complete paraplegia, sensory loss in the lower extremities, and bladder and rectal dysfunction. Gadolinium-enhanced magnetic resonance imaging showed enhancing spinal epidural lesions. A histopathological examination revealed dense lymphocyte and plasma-cell infiltration with fibrosis and increased IgG4-positive plasma cells, leading to the diagnosis of IgG4-RP. Although the serum IgG4 concentration was within the reference range, retrospective analysis of cerebrospinal fluid (CSF) showed a markedly elevated IgG4 concentration, with an increased IgG4 index and IgG4_Loc_. Glucocorticoid therapy resulted in radiological and neurological improvement. This case highlights the importance of considering IgG4-RP in patients with spinal HP even if serum IgG4 concentration is not elevated. Furthermore, CSF IgG4 measurement may contribute to the diagnosis of IgG4-RP, although further studies are required to establish its clinical utility.

## Introduction

Hypertrophic pachymeningitis (HP) is a rare disease characterized by thickening of the cranial and spinal dura mater caused by chronic inflammation. This disease presents with various symptoms, including headache, cranial nerve dysfunction, cerebellar ataxia, and myelopathy. IgG4-related disease (IgG4-RD) is a fibroinflammatory disease characterized by dense lymphoplasmacytic infiltration with IgG4-positive plasma cells and the development of organ swelling, mass, or nodule lesions [1,2]. Immunoglobulin G4-related hypertrophic pachymeningitis (IgG4-RP) is recognized as an uncommon cause of HP and represents a rare manifestation of IgG4-RD [3-5]. Although an elevated serum IgG4 concentration is included in the diagnostic criteria for IgG4-RD [6], serum IgG4 concentrations are not necessarily elevated in patients with IgG4-RP [5,7]. Consequently, histopathological evaluation is generally required for diagnosis, making the diagnosis of IgG4-RP challenging. Conversely, an elevated cerebrospinal fluid (CSF) IgG4 concentration, even in patients with normal serum IgG4 concentrations, may provide an important diagnostic clue to IgG4-RP. Early recognition through CSF IgG4 measurement may allow timely initiation of treatment, potentially leading to better neurological outcomes. Although an elevation of CSF IgG4 concentration may contribute to the diagnosis [7,8], reports describing CSF IgG4 concentration in IgG4-RP remain limited. In particular, reports of IgG4-RP presenting with severe paraplegia, despite serum IgG4 concentrations within the reference range and markedly elevated CSF IgG4 concentrations, are extremely rare. We report a rare case of paraplegia caused by IgG4-RP with a highly elevated CSF IgG4 concentration.

## Case presentation

A 78-year-old woman with a history of lumbar spinal canal stenosis was admitted to the referring hospital with low back pain for one week, difficulty in standing, and constipation for several days. The patient was taking celecoxib and mirogabalin. The patient had no history of autoimmune disease, and her family history was unremarkable. In the referring hospital, the patient showed bilateral lower-extremity motor and sensory impairment and an elevated serum C-reactive protein concentration. Over approximately one week, bilateral lower-extremity motor weakness and sensory deficits progressed, eventually resulting in complete paraplegia. Eleven days after admission to the referring hospital, the patient was referred to our hospital for further diagnostic evaluation and management.

On admission to our hospital, complete paraplegia with decreased sensation in all sensory modalities below the inguinal region was observed. The patient also presented with constipation and urinary retention, consistent with bladder and rectal dysfunction. The patient was classified as ASIA Impairment Scale grade B, and deep tendon reflexes were absent in both lower extremities. Laboratory data showed an elevated white blood cell count and C-reactive protein concentration (Table 1; Appendices).

**Table 1:** Laboratory findings on admission to our hospital. Abnormal values are shown in bold.

Complete blood count	Result	Units	Reference range
White blood cell	12,900	/µL	3,300-8,600
Neutrophils	85.5	%	38-74
Lymphocytes	7.4	%	16.5-49.5
Monocytes	5.9	%	2-10
Eosinophils	1.0	%	0-8.5
Basophils	0.2	%	0-2.5
Red blood cell	380 × 10^4^	/µL	386 × 10^4^-492 × 10^4^
Hemoglobin	10.8	g/dL	11.6-14.8
Hematocrit	31.9	%	35.1-44.4
Platelet	487 × 10³	/µL	158 × 10³-348 × 10³
Blood chemistry			
Sodium	126	mEq/L	138-145
Chloride	91	mEq/L	101-108
Potassium	4.6	mEq/L	3.6-4.8
Calcium	8.1	mg/dL	8.8-10.1
Phosphorus	3.3	mg/dL	2.7-4.6
Iron	12	µg/dL	40-188
C-reactive protein	8.17	mg/dL	0-0.14
Fasting blood glucose	162	mg/dL	70-109
Hemoglobin A1c	6.2	%	4.9-6.0
Vitamin B1	468	ng/mL	24-66
Vitamin B12	≥1,500	pg/mL	180-914
Folate	4.2	ng/mL	4≦
Immunology			
Immunoglobulin A	266	mg/dL	93-393
Immunoglobulin G	1450	mg/dL	861-1747
Immunoglobulin G4	110	mg/dL	11-121
Immunoglobulin M	108	mg/dL	50-269
Angiotensin-converting enzyme (ACE)	7.0	U/L	8.3-21.4
Lysozyme	7.4	µg/mL	5-10.2
Myeloperoxidase–antineutrophil cytoplasmic antibody (MPO-ANCA)	2.5	U/mL	0-3.4
Proteinase 3–antineutrophil cytoplasmic antibody (PR3-ANCA)	<1.0	U/mL	0-3.4
anti-Ro/SS-A antibody	<1.0	U/mL	0-10.0
anti-La/SS-B antibody	<1.0	U/mL	0-10.0
Soluble interleukin-2 receptor	1,452.7	U/mL	122-496
Infection serology			
Interferon-gamma release assay	(-)		

Additionally, the soluble interleukin-2 receptor concentration was increased. The serum IgG concentration was 1,450 mg/dL, and the IgG4 concentration was 110 mg/dL, both of which were within reference ranges. Myeloperoxidase anti-neutrophil cytoplasmic antibody, proteinase 3 anti-neutrophil cytoplasmic antibody, anti-Ro/SS-A antibody, anti-La/SS-B antibody, and the interferon-gamma release assay were negative. A CSF analysis showed mononuclear cell-predominant pleocytosis and elevated protein and decreased glucose concentrations (Table 2).

**Table 2:** Cerebrospinal fluid (CSF) analysis on admission to our hospital. Abnormal values are shown in bold.

	Result	Units	Reference range
Total cell count	213	/mm³	0-5
Mononuclear cells	151	/mm³	0-5
Polymorphonuclear cells	62	/mm³	0
Protein	1,798	mg/dL	10-40
Albumin	512.8	mg/dL	5-24
Glucose	13	mg/dL	50-75
(Simultaneous blood glucose)	113	mg/dL	73-109
Chloride	111	mmol/L	120-130

**Figure 1:**
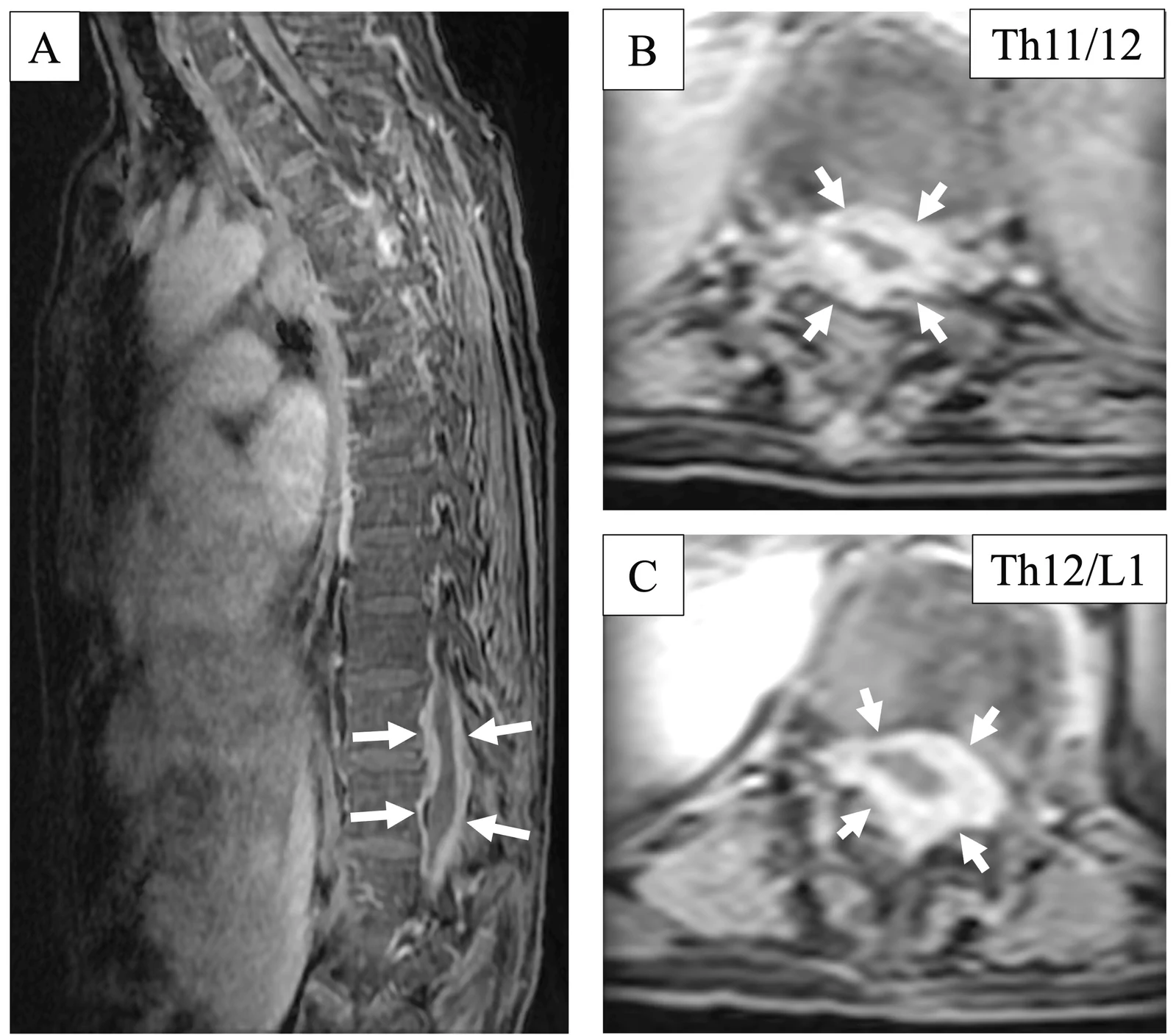
Gadolinium-enhanced MRI on admission to our hospital. (A) Contrast-enhanced sagittal T1-weighted image shows a flattened, contrast-enhancing mass in the spinal epidural space extending from the Th11 to L1 levels (white arrow). (B) Axial image shows an epidural mass compressing the spinal cord at the Th11/12 level (white arrow). (C) Similarly, spinal cord compression was observed at the Th12/L1 level (white arrow).

Gadolinium-enhanced spinal MRI demonstrated a flattened, contrast-enhancing mass in the spinal epidural space extending from the Th4 to Th6 levels and from the Th11 to L1 levels (Figure 1).

**Figure 2:**
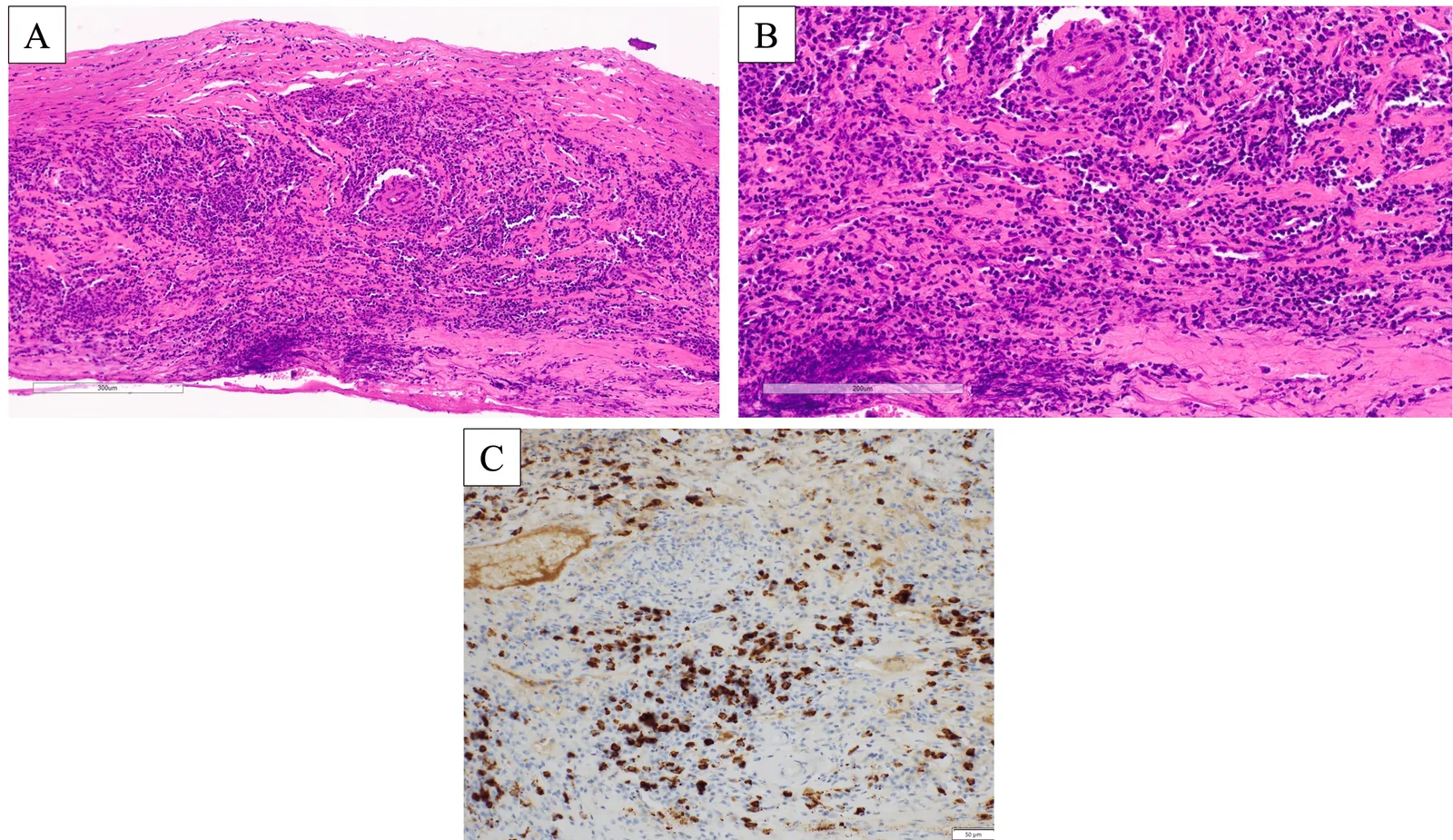
Pathological findings of the dura mater. (A) Hematoxylin and eosin staining shows irregular fibrous thickening. Scale bar, 300 µm. (B) A medium-power view of hematoxylin and eosin staining shows marked infiltration of lymphocytes and plasma cells. Scale bar, 200 µm. (C) Immunohistochemical staining for immunoglobulin G4 shows dense infiltration by more than 100 immunoglobulin G4-positive plasma cells per high-power field. Scale bar, 50 µm.

Because imaging findings alone were insufficient to establish the diagnosis, histopathological evaluation was considered necessary. In addition, given the severity of the neurological deficits and the need for early decompression, surgical intervention was deemed necessary. On hospital day 2 at our institution, laminectomy of Th10 to L1 was performed for spinal cord decompression, and a mass lesion in the epidural space that was tightly adherent to the dura mater was observed. Although maximal safe dissection was attempted, adequate decompression may not have been achieved. On hospital day 9, a histopathological evaluation of the mass lesion showed dense lymphocyte and plasma cell infiltration with fibrosis (Figure 2A-B). The number of IgG4-positive plasma cells was greater than 100 per high-power field (Figure 2C).

Accordingly, the patient fulfilled the clinical and radiological features and pathological diagnosis components of the diagnostic criteria [6]. However, because the serological criterion was not met, the diagnosis was classified as probable IgG4-RP in accordance with the 2020 revised comprehensive diagnostic criteria for IgG4-RD [6]. No other organ involvement was identified on additional imaging studies, including gadolinium-enhanced brain MRI.

**Figure 3:**
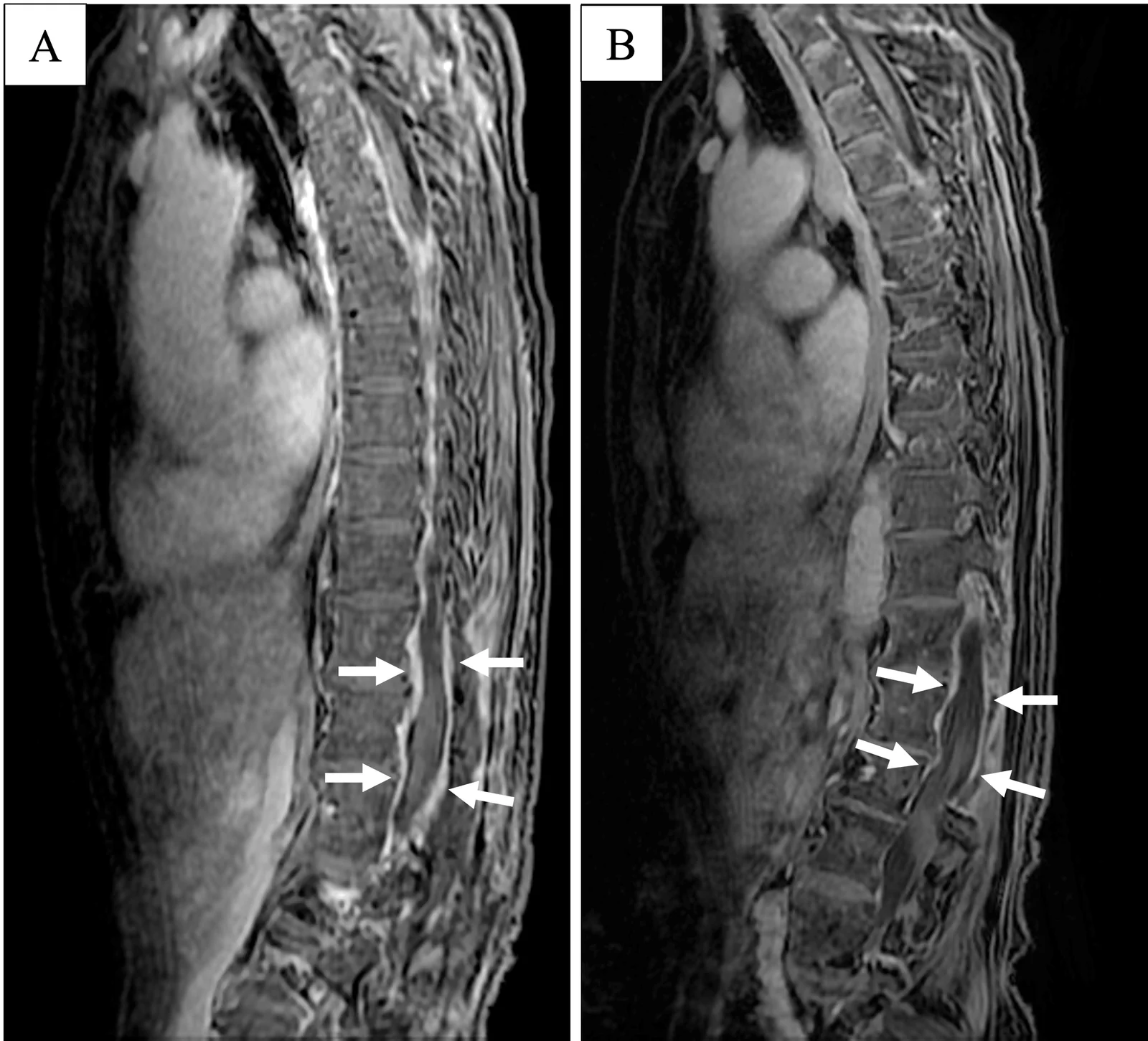
Follow-up gadolinium-enhanced MRI. (A) On day 2 after the initiation of intravenous hydrocortisone, a contrast-enhanced sagittal T1-weighted image shows a reduction in dural thickening (white arrow). (B) On day 34 after initiating treatment, contrast-enhanced MRI shows almost complete disappearance of the dural thickening (white arrow).

On hospital day 10, treatment was initiated with intravenous methylprednisolone at a dose of 1,000 mg/day. From the following day, voluntary contractions of the lower extremities and spontaneous movements of the toes were observed. On the same day, follow-up gadolinium-enhanced MRI showed a reduction in dural thickening (Figure 3A). On hospital day 14, therapy was switched to oral prednisolone at a dose of 40 mg/day. On hospital day 42, muscle strength in the lower extremities improved to at least grade 3 on manual muscle testing, corresponding to ASIA Impairment Scale grade D. On the same day, gadolinium-enhanced MRI showed almost complete disappearance of the dural thickening (Figure 3B). The patient was subsequently transferred to another hospital for continued rehabilitation while receiving oral prednisolone at a dose of 30 mg/day.

The measurement of CSF IgG4 was not performed initially because it is not covered by the Japanese national health insurance system and is therefore not routinely performed in clinical practice. However, after hospital transfer, additional analysis of the CSF was performed and showed a highly elevated IgG4 concentration of 96.8 mg/dL (Table 3). The IgG4 index and IgG4Loc were calculated as 4.29 and 76.0 [1,9], respectively, both of which were elevated. CSF IgG4 concentration was assessed using turbidimetric immunoassay (TIA; BioMajesty^TM^ JCA-BM8000 series; JEOL Ltd., Tokyo, Japan). The CSF sample used for IgG4 measurement was obtained at the admission, which had been appropriately preserved at -80℃ for potential retrospective testing. The reference range of CSF IgG4 concentration, IgG4 index, and IgG4Loc was cited from a previous report [8].

**Table 3:** CSF IgG4 parameters measured or calculated retrospectively using a sample obtained at admission. The IgG4 Index and IgG4Loc are both markers of intrathecal IgG4 synthesis. CSF IgG4 concentrations >2.27 mg/dL were reported to show 100% sensitivity, and an IgG4Loc >0.47 was reported to show 100% sensitivity and specificity in differentiating IgG4-RP from other types of HP [8]. HP: hypertrophic pachymeningitis; CSF: cerebrospinal fluid; IgG4-RP: Immunoglobulin G4-related hypertrophic pachymeningitis.

	Result	Units	Reference range
CSF IgG4	96.8	mg/dL	0.01-0.33
IgG4 index	4.29		0.25-2.11
IgG4_Loc_	4.53		0

The patient was transferred to a rehabilitation hospital while receiving oral prednisolone at a dose of 30 mg/day. Prednisolone was gradually tapered, reaching 10 mg/day approximately five months after transfer, at which time the patient was discharged. At discharge, the patient was able to ambulate using a non-wheeled walker and remained classified as ASIA Impairment Scale grade D. Approximately two weeks after discharge, the patient developed recurrent low back pain and worsening lower-extremity weakness, eventually becoming unable to maintain a standing position even with the use of a walker. Spinal MRI demonstrated progression of dural thickening, and the patient was diagnosed with a relapse of IgG4-RP. Intravenous methylprednisolone at a dose of 1,000 mg/day was administered, followed by an increase in oral prednisolone to 20 mg/day. One month later, azathioprine at a dose of 100 mg/day was added while prednisolone was tapered to 10 mg/day. Since then, the patient has remained neurologically stable with ASIA Impairment Scale grade D, and the doses of prednisolone and azathioprine have been adjusted according to her clinical course. No additional organ involvement developed during follow-up.

## Discussion

In the present case, the patient developed paraplegia with thickening of the spinal dura mater. The differential diagnoses included anti-neutrophil cytoplasmic antibody (ANCA)-related vasculitis, Bechet’s disease, Sjogren’s syndrome, sarcoidosis, tuberculosis, other infections, Erdheim-Chester disease, Rosai-Dorfman disease, malignant lymphoma, and IgG4-RP. Based on serological findings, ANCA-related vasculitis and Sjogren’s syndrome were considered unlikely because disease-specific autoantibodies were negative. Tuberculosis was also ruled out because of the negative interferon-gamma release assay result. There were no typical symptoms or lesions suggestive of Bechet’s disease. Sarcoidosis was considered unlikely because of the absence of lymphadenopathy, multiple organ lesions, and an elevated serum angiotensin-converting enzyme concentration. There were no typical clinical features suggestive of Erdheim-Chester disease, such as bone pain, xanthelasma, exophthalmos, ataxia, or diabetes insipidus. Malignant lymphoma required the most careful differentiation because of the elevated soluble interleukin-2 receptor concentration. Although malignant lymphoma could not be excluded until surgical biopsy, a histopathological evaluation ruled it out because there was no evidence of monoclonal lymphoid proliferation or a monotonous proliferation of atypical lymphoid cells. Rosai-Dorfman disease was also considered in the differential diagnosis. However, the absence of lymphadenopathy and the lack of emperipolesis on histopathological evaluation argued against this diagnosis. Although the CSF analysis showed marked pleocytosis, elevated protein, and low glucose suggestive of an infectious etiology, CSF culture was not performed. However, the histopathological findings were not consistent with infection and instead supported the diagnosis of IgG4-RP, demonstrating dense lymphoplasmacytic infiltration with fibrosis and abundant IgG4-positive plasma cells. After the diagnosis, high-dose glucocorticoid therapy was immediately initiated, which resulted in rapid improvement of the dural thickening observed on MRI. Lower-extremity muscle strength also gradually improved, which indicated a favorable response to glucocorticoid therapy.

HP is a chronic inflammatory disease that causes thickening of the dura mater and various neurological symptoms. The symptoms of HP depend on the anatomical distribution of the lesions, including headache, cranial nerve dysfunction, cerebellar ataxia, and myelopathy. HP is classified as idiopathic or secondary according to whether a specific cause is identified. Common causes of secondary HP are tumors, infections, and autoimmune disease, including IgG4-RD, ANCA-related vasculitis, and Sjogren’s syndrome [3]. According to a nationwide survey in Japan [3], the most frequent cause of HP is ANCA-related vasculitis, accounting for 30.2% of cases, whereas IgG4-RD is rare, comprising only 8.8%.

IgG4-RD is a fibroinflammatory disease characterized by dense infiltration of lymphocytes and IgG4-positive plasma cells and organ swelling, mass, or nodule lesions [1,2]. The most frequent manifestations of IgG4-RD are pancreatitis, sialadenitis, nephritis, dacryoadenitis, and periaortitis [10]. IgG4-RP is an extremely rare type of IgG4-RD, and IgG4-RP is typically recognized as enhancing dural thickening on contrast-enhanced MRI. IgG4-RP has been reported to occur in only 2.4-4.1% of patients with IgG4-RD, indicating its rarity [4,5]. Male predominance is characteristic of this disease, with a male-to-female ratio of 1:0.17 [3]. According to previous studies, including a literature review, the distribution of lesions has been reported to involve the cranial dura in 82-100% and the spinal dura in 0-27% of cases [2,7], indicating that spinal involvement is rare. To diagnose IgG4-RP, there are no organ-specific criteria, although diagnostic criteria for autoimmune pancreatitis, IgG4-related sclerosing cholangitis, dacryoadenitis, and sialadenitis have been published [11-13]. Therefore, comprehensive diagnostic criteria for IgG4-RD are adopted to diagnose IgG4-RP [6]. The criteria for IgG4-RD consist of clinical and radiological features, elevation of serum IgG4 concentrations, and pathological findings. Serum IgG4 concentrations lack sufficient sensitivity and specificity. Therefore, clinical and radiological features and pathological findings are more contributive for the diagnosis in these criteria. Previous reports, including a review article, showed that an elevation in serum IgG4 concentrations was observed in only 50-55% of cases, even when limited to IgG4-RP [5,7]. However, one report showed that CSF IgG4 concentrations exceeded 10 times the upper limit of normal in all four patients studied [7]. Della-Torre et al. evaluated the diagnostic value of the CSF IgG4 concentration, IgG4 index, and IgG4Loc [8]. The IgG4 index and IgG4Loc are calculated using the same formulas as the IgG index and IgGLoc [1,9]. They reported that CSF IgG4 concentrations >2.27 mg/dL showed 100% sensitivity, and an IgG4Loc >0.47 showed 100% sensitivity and specificity in differentiating IgG4-RP from other types of HP. In the present case, the CSF IgG4 concentration was 96.8 mg/dL, the IgG4Loc was calculated to be 76.0, and the IgG4 index was calculated to be 4.29 (Table 3). These findings were consistent with previously reported cut-off values [8]. Therefore, the CSF IgG4 concentration and IgG4Loc are considered to have potential diagnostic value, although further validation is required. Regarding pathological features, dense infiltration of lymphocytes/plasma cells with fibrosis, increased IgG4-positive plasma cells, storiform fibrosis, and obliterative phlebitis are characteristic findings of IgG4-RD. Among these, storiform fibrosis and obliterative phlebitis are considered particularly specific and useful for diagnosing IgG4-RD [14]. Although some pathological differences among organs are known in IgG4-RD, organ-specific pathological features of IgG4-RP remain unclear, representing an important subject for future investigation. The clinical symptoms of IgG4-RP are generally similar to other types of HP, including headache, cranial nerve dysfunction, paralysis and paresthesia of the extremities, and seizures. Paraplegia has also been reported as a symptom of IgG4-RP [15,16], but its frequency remains unclear. The treatment of IgG4-RP is typically guided by recommendations for other IgG4-RD manifestations because no disease-specific standard has been established. Glucocorticoids are recommended as the first-line therapy in patients with IgG4-RD [17,18]. In cases of IgG4-RD in which glucocorticoid therapy is insufficient, rituximab and immunosuppressive drugs, including thiopurines, mycophenolate mofetil, methotrexate, or calcineurin inhibitors, are recommended [17,18]. Similarly, in previously reported cases of IgG4-RP [1], glucocorticoids were the most frequently used therapy, although rituximab, immunosuppressive drugs, and surgical management have also been reported. In the present case, the patient showed an excellent response to glucocorticoid therapy, which suggested that earlier initiation of glucocorticoids before histopathological confirmation might have resulted in a more favorable neurological outcome. Surgical decompression may be effective in patients with severe or progressive neurological deficits caused by spinal cord compression, but adjunctive therapy with glucocorticoids and/or immunosuppressive agents is generally required to prevent recurrence [19]. In the present case, it remains uncertain whether earlier initiation of glucocorticoid therapy could have achieved similar or better outcomes without surgery. In cases such as the present case, in which decompression may have been insufficient with laminectomy alone, dural resection and duraplasty could be considered [20]. IgG4-RP can present with a wide variety of clinical symptoms, including paraplegia, depending on the location of the lesions. Therefore, IgG4-RP should be included in the differential diagnosis when thickening of the dura mater is identified. Considering that IgG4-RP is an extremely rare disease, neither organ-specific diagnostic criteria nor treatment strategies have been established. Regarding the diagnosis of IgG4-RP, this case raises the possibility that elevated CSF IgG4 concentration may provide supportive diagnostic information in conjunction with histopathological findings, although this hypothesis requires confirmation in larger studies. We speculate that the perineural space surrounding the spinal cord is more limited than the space around intracranial lesions in IgG4-RP. Therefore, spinal lesions may be more likely to cause rapidly progressive and severe neurological deficits owing to mechanical compression, as in the present case. An early diagnosis and prompt initiation of glucocorticoid therapy may lead to neurological improvement, highlighting the particular importance of timely intervention in cases of IgG4-RP with spinal involvement.

## Conclusions

IgG4-RP should be considered in the differential diagnosis of spinal HP, even in patients with normal serum IgG4 concentrations. Because spinal involvement may cause rapidly progressive neurological deficits, early recognition and prompt treatment are important to reduce the risk of irreversible neurological impairment.

Although histopathological examination remains the diagnostic gold standard for IgG4-RP, this case also suggests that CSF IgG4 concentration may provide additional diagnostic information, particularly when serum IgG4 concentrations are within the reference range. CSF IgG4 concentration may serve as an adjunctive biomarker to support the diagnosis rather than replace histopathological confirmation. Because this report describes a single case, these findings should be considered hypothesis-generating. Further studies are needed to establish its clinical utility.

## Appendices

Appendix A

**Table 4:** Supplementary laboratory findings on admission to our hospital. Abnormal values are shown in bold.

Blood chemistry	Result	Units	Reference range
Total protein	6.4	g/dL	6.6-8.1
Total bilirubin	0.3	mg/dL	0.4-1.5
Aspartate aminotransferase	14	U/L	13-30
Alanine aminotransferase	11	U/L	7-23
Alkaline phosphatase	107	U/L	38-113
Gamma-glutamyl transpeptidase	19	U/L	9-32
Lactate dehydrogenase	139	U/L	124-222
Albumin	2.5	g/dL	4.1-5.1
Blood urea nitrogen	7.0	mg/dL	8-20
Creatinine	0.4	mg/dL	0.46-0.79
Estimated glomerular filtration rate	112	mL/min/1.73m^2^	60≦
